# Morpholinium hydrogen chloranilate methanol monosolvate

**DOI:** 10.1107/S1600536811047891

**Published:** 2011-11-16

**Authors:** Kazuma Gotoh, Yuki Tahara, Hiroyuki Ishida

**Affiliations:** a Department of Chemistry, Faculty of Science, Okayama University, Okayama 700-8530, Japan

## Abstract

In the crystal structure of the title compound, C_4_H_10_NO^+^·C_6_HCl_2_O_4_^−^·CH_4_O, the components are held together by bifurcated O—H⋯(O,O), O—H⋯(O,Cl) and N—H⋯(O,O) hydrogen bonds into a centrosymmetric 2+2+2 aggregate. The aggregates are further connected by another bifurcated N—H⋯(O, O) hydrogen bond, forming a double-tape structure along the *b* axis. A weak C—H⋯O inter­action is observed between the tapes.

## Related literature

For a related structure, see: Ishida & Kashino (1999 ▶). For ^35^Cl nuclear quadrupole resonance studies on proton-transfer in chloranilic acid–organic base systems, see: Ikeda *et al.* (2005 ▶); Asaji, Hoshino *et al.* (2010 ▶); Asaji, Seliger *et al.* (2010 ▶).
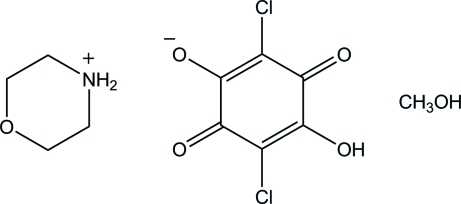


## Experimental

### 

#### Crystal data

C_4_H_10_NO^+^·C_6_HCl_2_O_4_^−^·CH_4_O
*M* *_r_* = 328.15
Triclinic, 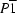

*a* = 9.11845 (17) Å
*b* = 9.39881 (17) Å
*c* = 9.96935 (18) Å
α = 107.8089 (7)°
β = 107.5510 (7)°
γ = 110.2398 (7)°
*V* = 679.25 (2) Å^3^
*Z* = 2
Mo *K*α radiation
μ = 0.50 mm^−1^
*T* = 170 K
0.45 × 0.41 × 0.30 mm

#### Data collection

Rigaku R-AXIS RAPID II diffractometer
Absorption correction: numerical (*NUMABS*; Higashi, 1999 ▶) *T*_min_ = 0.817, *T*_max_ = 0.860
17817 measured reflections
3928 independent reflections
3636 reflections with *I* > 2σ(*I*)
*R*_int_ = 0.025

#### Refinement

*R*[*F* ^2^ > 2σ(*F* ^2^)] = 0.027
*wR*(*F* ^2^) = 0.077
*S* = 1.08
3928 reflections
197 parameters
H atoms treated by a mixture of independent and constrained refinement
Δρ_max_ = 0.51 e Å^−3^
Δρ_min_ = −0.28 e Å^−3^

### 

Data collection: *PROCESS-AUTO* (Rigaku/MSC, 2004 ▶); cell refinement: *PROCESS-AUTO*; data reduction: *CrystalStructure* (Rigaku/MSC, 2004 ▶); program(s) used to solve structure: *SHELXS97* (Sheldrick, 2008 ▶); program(s) used to refine structure: *SHELXL97* (Sheldrick, 2008 ▶); molecular graphics: *ORTEP-3* (Farrugia, 1997 ▶); software used to prepare material for publication: *SHELXL97* and *PLATON* (Spek, 2009 ▶).

## Supplementary Material

Crystal structure: contains datablock(s) General, I. DOI: 10.1107/S1600536811047891/lh5377sup1.cif

Structure factors: contains datablock(s) I. DOI: 10.1107/S1600536811047891/lh5377Isup2.hkl

Supplementary material file. DOI: 10.1107/S1600536811047891/lh5377Isup3.cml

Additional supplementary materials:  crystallographic information; 3D view; checkCIF report

## Figures and Tables

**Table 1:** Hydrogen-bond geometry (Å, °)

*D*—H⋯*A*	*D*—H	H⋯*A*	*D*⋯*A*	*D*—H⋯*A*
N1—H1*A*⋯O3	0.878 (18)	2.391 (18)	3.0069 (12)	127.5 (16)
N1—H1*A*⋯O3^i^	0.878 (18)	2.180 (19)	2.9255 (13)	142.5 (16)
N1—H1*B*⋯O1^ii^	0.852 (19)	2.170 (19)	2.9207 (14)	146.9 (17)
N1—H1*B*⋯O4^ii^	0.852 (19)	2.233 (19)	2.9277 (14)	138.7 (16)
O2—H2⋯O3	0.82 (2)	2.26 (2)	2.6605 (12)	110.6 (16)
O2—H2⋯O6	0.82 (2)	1.79 (2)	2.5564 (13)	153.4 (19)
O6—H6⋯Cl2^i^	0.742 (19)	2.761 (19)	3.3342 (9)	136.0 (18)
O6—H6⋯O3^i^	0.742 (19)	2.119 (19)	2.7812 (12)	149 (2)
C8—H8*A*⋯O2^iii^	0.99	2.51	3.4115 (15)	152

Symmetry codes: (i) 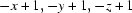; (ii) 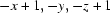; (iii) 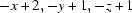.

**Fig. 1.**
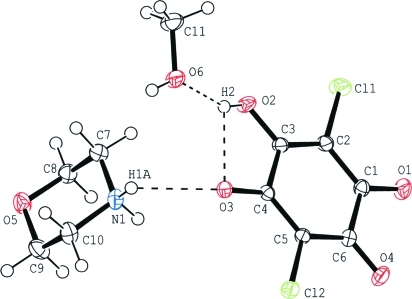
The molecular structure of the title compound, with the atom-labeling. Displacement ellipsoids of non-H atoms are drawn at the 50% probability level. The dashed lines indicate the O—H···O and N—H···O hydrogen bonds.

**Fig. 2.**
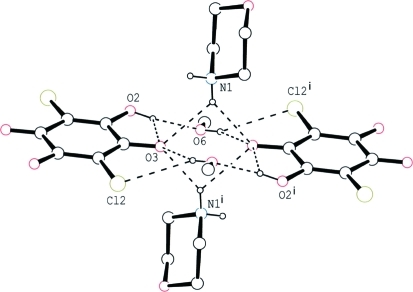
A view of the centrosymmetric 2 + 2+2 aggregate of the title compound. The O—H···(O, O), O—H···(O, Cl) and N—H···(O, O) hydrogen bonds are indicated by dashed lines. H atoms not involved in the hydrogen bonds have been omitted. [Symmetry code: (i) -x + 1, -y + 1, -z + 1.]

**Fig. 3.**
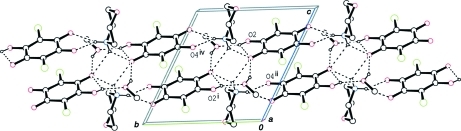
A partial packing view of the title compound, showing the double-tape structure. H atoms not involved in the hydrogen bonds have been omitted. [Symmetry codes: (i) -x + 1, -y + 1, -z + 1; (ii) -x + 1, -y, -z + 1; (iii) x, y + 1, z.]

## supplementary crystallographic information

### Comment

The title compound was accidentally obtained in the preparation of morpholinium hydrogen chloranilate (Ishida & Kashino, 1999), C_4_H_10_NO^+^.C_6_HCl_2_O_4_^-^, which is an interesting model compound for investigating proton transfer in the hydrogen bond systems (Ikeda *et al.*, 2005; Asaji, Hoshino *et al.*, 2010; Asaji, Seliger *et al.*, 2010).

In the title compound, the three components (Fig. 1) are held together by bifurcated O—H···(O, O), O—H···(O, Cl) and N—H···(O, O) hydrogen bonds [O2—H2···(O3, O6), O6—H6···(O3^i^, Cl2^i^) and N1—H1A···(O3, O3^i^); symmetry code in Table 1] into a centrosymmetric 2 + 2+2 aggregate (Fig. 2). The aggregates are connected by another N—H···(O, O) hydrogen bond between the cation and the anion [N1—H1B···(O1^ii^, O4^ii^), symmetry code in Table 1], forming a double-tape structure along the *b* axis (Fig. 3). The tapes are further linked a weak C—H···O interaction, forming a three-dimensional network.

### Experimental

Single crystals were obtained by slow evaporation from a methanol solution (50 ml) of chloranilic acid (0.102 g) and morpholine (0.044 g) at room temperature.

### Refinement

C-bound H atoms were positioned geometrically (C—H = 0.98 or 0.99 Å) and refined as riding, with *U*_iso_(H) = 1.2*U*_eq_(C) or 1.5*U*_eq_(methyl C). The O– and N-bound H atoms were found in a difference Fourier map and refined freely. The refined distances are O—H = 0.82 (2) and 0.742 (19) Å, and N—H = 0.852 (19) and 0.878 (18) Å.

### Crystal data

**Table:**

C_4_H_10_NO^+^·C_6_HCl_2_O_4_^−^·CH_4_O	*Z* = 2
*M**_r_* = 328.15	*F*(000) = 340.00
Triclinic, *P*1	*D*_x_ = 1.604 Mg m^−^^3^
Hall symbol: -P 1	Mo *K*α radiation, λ = 0.71075 Å
*a* = 9.11845 (17) Å	Cell parameters from 16546 reflections
*b* = 9.39881 (17) Å	θ = 3.6–30.1°
*c* = 9.96935 (18) Å	µ = 0.50 mm^−^^1^
α = 107.8089 (7)°	*T* = 170 K
β = 107.5510 (7)°	Block, brown
γ = 110.2398 (7)°	0.45 × 0.41 × 0.30 mm
*V* = 679.25 (2) Å^3^	

### Data collection

**Table:**

Rigaku R-AXIS RAPID II diffractometer	3636 reflections with *I* > 2σ(*I*)
Detector resolution: 10.00 pixels mm^-1^	*R*_int_ = 0.025
ω scans	θ_max_ = 30.0°
Absorption correction: numerical (*NUMABS*; Higashi, 1999)	*h* = −12→12
*T*_min_ = 0.817, *T*_max_ = 0.860	*k* = −13→13
17817 measured reflections	*l* = −14→14
3928 independent reflections	

### Refinement

**Table:**

Refinement on *F*^2^	Primary atom site location: structure-invariant direct methods
Least-squares matrix: full	Secondary atom site location: difference Fourier map
*R*[*F*^2^ > 2σ(*F*^2^)] = 0.027	Hydrogen site location: inferred from neighbouring sites
*wR*(*F*^2^) = 0.077	H atoms treated by a mixture of independent and constrained refinement
*S* = 1.08	*w* = 1/[σ^2^(*F*_o_^2^) + (0.0459*P*)^2^ + 0.1482*P*] where *P* = (*F*_o_^2^ + 2*F*_c_^2^)/3
3928 reflections	(Δ/σ)_max_ = 0.001
197 parameters	Δρ_max_ = 0.51 e Å^−^^3^
0 restraints	Δρ_min_ = −0.28 e Å^−^^3^

### Special details

**Table:**

Geometry. All e.s.d.'s (except the e.s.d. in the dihedral angle between two l.s. planes) are estimated using the full covariance matrix. The cell e.s.d.'s are taken into account individually in the estimation of e.s.d.'s in distances, angles and torsion angles; correlations between e.s.d.'s in cell parameters are only used when they are defined by crystal symmetry. An approximate (isotropic) treatment of cell e.s.d.'s is used for estimating e.s.d.'s involving l.s. planes.
Refinement. Refinement of *F*^2^ against ALL reflections. The weighted *R*-factor *wR* and goodness of fit *S* are based on *F*^2^, conventional *R*-factors *R* are based on *F*, with *F* set to zero for negative *F*^2^. The threshold expression of *F*^2^ > σ(*F*^2^) is used only for calculating *R*-factors(gt) *etc*. and is not relevant to the choice of reflections for refinement. *R*-factors based on *F*^2^ are statistically about twice as large as those based on *F*, and *R*- factors based on ALL data will be even larger.

### Fractional atomic coordinates and isotropic or equivalent isotropic displacement parameters (Å^2^)

**Table:**

	*x*	*y*	*z*	*U*_iso_*/*U*_eq_	
Cl1	1.01275 (3)	0.30381 (3)	0.86968 (3)	0.02809 (7)	
Cl2	0.20309 (3)	0.06793 (3)	0.53010 (3)	0.02191 (7)	
O1	0.71379 (10)	0.00021 (10)	0.82390 (9)	0.02629 (15)	
O2	0.84595 (9)	0.45763 (9)	0.70404 (9)	0.02291 (14)	
O3	0.50528 (8)	0.35907 (8)	0.55923 (8)	0.01801 (13)	
O4	0.37147 (9)	−0.10742 (9)	0.67309 (9)	0.02464 (15)	
O5	0.58600 (10)	0.31839 (10)	−0.00604 (8)	0.02641 (15)	
O6	0.80802 (9)	0.70163 (9)	0.66544 (10)	0.02655 (16)	
N1	0.52494 (12)	0.35047 (11)	0.26136 (10)	0.02167 (16)	
C1	0.67022 (12)	0.08381 (11)	0.76387 (10)	0.01785 (16)	
C2	0.79228 (11)	0.23463 (11)	0.77003 (11)	0.01801 (16)	
C3	0.73479 (11)	0.32094 (10)	0.70050 (10)	0.01635 (15)	
C4	0.54246 (11)	0.26734 (10)	0.61815 (9)	0.01450 (15)	
C5	0.42266 (11)	0.12427 (11)	0.61394 (10)	0.01582 (15)	
C6	0.47242 (11)	0.02420 (11)	0.67825 (10)	0.01687 (16)	
C7	0.70652 (13)	0.42900 (12)	0.27855 (11)	0.02421 (18)	
H7A	0.7887	0.4300	0.3706	0.029*	
H7B	0.7451	0.5482	0.2962	0.029*	
C8	0.70673 (13)	0.32531 (13)	0.12851 (12)	0.02324 (18)	
H8A	0.8263	0.3774	0.1376	0.028*	
H8B	0.6745	0.2081	0.1150	0.028*	
C9	0.41236 (13)	0.23401 (14)	−0.02607 (12)	0.0271 (2)	
H9A	0.3830	0.1173	−0.0391	0.033*	
H9B	0.3285	0.2242	−0.1231	0.033*	
C10	0.39445 (13)	0.33079 (14)	0.11517 (12)	0.02451 (19)	
H10A	0.4149	0.4445	0.1240	0.029*	
H10B	0.2742	0.2678	0.1012	0.029*	
C11	0.98148 (13)	0.84290 (14)	0.75821 (14)	0.0311 (2)	
H11A	1.0434	0.8324	0.8508	0.047*	
H11B	0.9754	0.9493	0.7933	0.047*	
H11C	1.0448	0.8441	0.6943	0.047*	
H1A	0.520 (2)	0.414 (2)	0.3435 (19)	0.036 (4)*	
H1B	0.4968 (19)	0.252 (2)	0.2566 (17)	0.032 (4)*	
H2	0.801 (2)	0.512 (2)	0.675 (2)	0.052 (5)*	
H6	0.750 (2)	0.718 (2)	0.609 (2)	0.041 (4)*	

### Atomic displacement parameters (Å^2^)

**Table:**

	*U*^11^	*U*^22^	*U*^33^	*U*^12^	*U*^13^	*U*^23^
Cl1	0.01597 (11)	0.02532 (12)	0.03726 (14)	0.00939 (9)	0.00314 (9)	0.01727 (10)
Cl2	0.01479 (10)	0.02700 (12)	0.02709 (12)	0.01005 (8)	0.00953 (8)	0.01609 (9)
O1	0.0255 (3)	0.0270 (3)	0.0347 (4)	0.0155 (3)	0.0119 (3)	0.0222 (3)
O2	0.0153 (3)	0.0180 (3)	0.0339 (4)	0.0068 (2)	0.0065 (3)	0.0162 (3)
O3	0.0178 (3)	0.0170 (3)	0.0206 (3)	0.0095 (2)	0.0066 (2)	0.0109 (2)
O4	0.0229 (3)	0.0235 (3)	0.0338 (4)	0.0104 (3)	0.0144 (3)	0.0198 (3)
O5	0.0241 (3)	0.0382 (4)	0.0223 (3)	0.0151 (3)	0.0135 (3)	0.0172 (3)
O6	0.0189 (3)	0.0227 (3)	0.0357 (4)	0.0082 (3)	0.0056 (3)	0.0194 (3)
N1	0.0300 (4)	0.0218 (4)	0.0196 (4)	0.0145 (3)	0.0139 (3)	0.0120 (3)
C1	0.0194 (4)	0.0179 (4)	0.0189 (4)	0.0104 (3)	0.0084 (3)	0.0103 (3)
C2	0.0146 (3)	0.0170 (4)	0.0213 (4)	0.0081 (3)	0.0051 (3)	0.0098 (3)
C3	0.0149 (3)	0.0146 (3)	0.0176 (4)	0.0069 (3)	0.0054 (3)	0.0073 (3)
C4	0.0151 (3)	0.0144 (3)	0.0138 (3)	0.0080 (3)	0.0058 (3)	0.0061 (3)
C5	0.0136 (3)	0.0175 (4)	0.0174 (4)	0.0079 (3)	0.0068 (3)	0.0091 (3)
C6	0.0189 (4)	0.0177 (4)	0.0178 (4)	0.0099 (3)	0.0098 (3)	0.0097 (3)
C7	0.0239 (4)	0.0213 (4)	0.0209 (4)	0.0078 (3)	0.0067 (3)	0.0091 (3)
C8	0.0207 (4)	0.0261 (4)	0.0258 (4)	0.0119 (3)	0.0115 (4)	0.0137 (4)
C9	0.0212 (4)	0.0362 (5)	0.0194 (4)	0.0123 (4)	0.0092 (4)	0.0096 (4)
C10	0.0269 (4)	0.0327 (5)	0.0238 (4)	0.0191 (4)	0.0145 (4)	0.0159 (4)
C11	0.0194 (4)	0.0250 (5)	0.0430 (6)	0.0080 (4)	0.0068 (4)	0.0192 (4)

### Geometric parameters (Å, °)

**Table:**

Cl1—C2	1.7168 (9)	C2—C3	1.3536 (11)
Cl2—C5	1.7246 (8)	C3—C4	1.5069 (11)
O1—C1	1.2221 (11)	C4—C5	1.3874 (11)
O2—C3	1.3148 (10)	C5—C6	1.4107 (11)
O2—H2	0.825 (19)	C7—C8	1.5161 (13)
O3—C4	1.2630 (10)	C7—H7A	0.9900
O4—C6	1.2349 (11)	C7—H7B	0.9900
O5—C9	1.4211 (12)	C8—H8A	0.9900
O5—C8	1.4219 (12)	C8—H8B	0.9900
O6—C11	1.4260 (12)	C9—C10	1.5148 (13)
O6—H6	0.740 (17)	C9—H9A	0.9900
N1—C10	1.4870 (12)	C9—H9B	0.9900
N1—C7	1.4904 (13)	C10—H10A	0.9900
N1—H1A	0.876 (16)	C10—H10B	0.9900
N1—H1B	0.853 (16)	C11—H11A	0.9800
C1—C2	1.4373 (12)	C11—H11B	0.9800
C1—C6	1.5413 (12)	C11—H11C	0.9800
			
C3—O2—H2	113.5 (13)	C8—C7—H7A	110.0
C9—O5—C8	109.92 (7)	N1—C7—H7B	110.0
C11—O6—H6	111.7 (13)	C8—C7—H7B	110.0
C10—N1—C7	111.68 (7)	H7A—C7—H7B	108.4
C10—N1—H1A	109.0 (10)	O5—C8—C7	110.97 (8)
C7—N1—H1A	109.8 (10)	O5—C8—H8A	109.4
C10—N1—H1B	108.2 (10)	C7—C8—H8A	109.4
C7—N1—H1B	109.5 (10)	O5—C8—H8B	109.4
H1A—N1—H1B	108.6 (14)	C7—C8—H8B	109.4
O1—C1—C2	123.92 (8)	H8A—C8—H8B	108.0
O1—C1—C6	117.69 (8)	O5—C9—C10	110.93 (8)
C2—C1—C6	118.39 (7)	O5—C9—H9A	109.5
C3—C2—C1	120.81 (8)	C10—C9—H9A	109.5
C3—C2—Cl1	120.88 (7)	O5—C9—H9B	109.5
C1—C2—Cl1	118.31 (6)	C10—C9—H9B	109.5
O2—C3—C2	120.97 (8)	H9A—C9—H9B	108.0
O2—C3—C4	117.02 (7)	N1—C10—C9	109.27 (8)
C2—C3—C4	122.00 (8)	N1—C10—H10A	109.8
O3—C4—C5	125.78 (8)	C9—C10—H10A	109.8
O3—C4—C3	116.12 (7)	N1—C10—H10B	109.8
C5—C4—C3	118.10 (7)	C9—C10—H10B	109.8
C4—C5—C6	123.01 (8)	H10A—C10—H10B	108.3
C4—C5—Cl2	118.77 (6)	O6—C11—H11A	109.5
C6—C5—Cl2	118.20 (6)	O6—C11—H11B	109.5
O4—C6—C5	125.85 (8)	H11A—C11—H11B	109.5
O4—C6—C1	116.53 (8)	O6—C11—H11C	109.5
C5—C6—C1	117.61 (7)	H11A—C11—H11C	109.5
N1—C7—C8	108.60 (8)	H11B—C11—H11C	109.5
N1—C7—H7A	110.0		
			
O1—C1—C2—C3	179.84 (9)	C3—C4—C5—Cl2	176.39 (6)
C6—C1—C2—C3	−0.87 (13)	C4—C5—C6—O4	−177.30 (9)
O1—C1—C2—Cl1	−0.89 (13)	Cl2—C5—C6—O4	4.08 (13)
C6—C1—C2—Cl1	178.40 (6)	C4—C5—C6—C1	3.11 (12)
C1—C2—C3—O2	−179.07 (8)	Cl2—C5—C6—C1	−175.50 (6)
Cl1—C2—C3—O2	1.68 (13)	O1—C1—C6—O4	−1.84 (12)
C1—C2—C3—C4	1.85 (13)	C2—C1—C6—O4	178.82 (8)
Cl1—C2—C3—C4	−177.40 (6)	O1—C1—C6—C5	177.79 (8)
O2—C3—C4—O3	0.18 (11)	C2—C1—C6—C5	−1.55 (12)
C2—C3—C4—O3	179.29 (8)	C10—N1—C7—C8	−53.98 (10)
O2—C3—C4—C5	−179.49 (8)	C9—O5—C8—C7	−62.82 (10)
C2—C3—C4—C5	−0.38 (12)	N1—C7—C8—O5	58.10 (10)
O3—C4—C5—C6	178.14 (8)	C8—O5—C9—C10	62.10 (11)
C3—C4—C5—C6	−2.22 (12)	C7—N1—C10—C9	53.70 (11)
O3—C4—C5—Cl2	−3.25 (12)	O5—C9—C10—N1	−57.15 (11)

### Hydrogen-bond geometry (Å, °)

**Table:**

*D*—H···*A*	*D*—H	H···*A*	*D*···*A*	*D*—H···*A*
N1—H1A···O3	0.878 (18)	2.391 (18)	3.0069 (12)	127.5 (16)
N1—H1A···O3^i^	0.878 (18)	2.180 (19)	2.9255 (13)	142.5 (16)
N1—H1B···O1^ii^	0.852 (19)	2.170 (19)	2.9207 (14)	146.9 (17)
N1—H1B···O4^ii^	0.852 (19)	2.233 (19)	2.9277 (14)	138.7 (16)
O2—H2···O3	0.82 (2)	2.26 (2)	2.6605 (12)	110.6 (16)
O2—H2···O6	0.82 (2)	1.79 (2)	2.5564 (13)	153.4 (19)
O6—H6···Cl2^i^	0.742 (19)	2.761 (19)	3.3342 (9)	136.0 (18)
O6—H6···O3^i^	0.742 (19)	2.119 (19)	2.7812 (12)	149 (2)
C8—H8A···O2^iii^	0.99	2.51	3.4115 (15)	152

Symmetry codes: (i) −*x*+1, −*y*+1, −*z*+1; (ii) −*x*+1, −*y*, −*z*+1; (iii) −*x*+2, −*y*+1, −*z*+1.
